# A Clinical Tool for Diagnosis of Isolated Torsion of the Right Fallopian Tube in a Virgin Girl

**Published:** 2020

**Authors:** Lialios A Georgios, Skoufi I Georgia, Tsagkoulis K Matthaios

**Affiliations:** 1- Department of Obstetrics and Gynaecology, University Hospital of Larisa, Mezourlo, Greece; 2- Occupational Medicine Office, University Hospital of Larisa, Mezourlo, Greece

**Keywords:** Fallopian tube, Isolated tubal torsion, Laparoscopy, Salpingectomy

## Abstract

**Background:**

Isolated tubal torsion is a rare condition that its management remains a challenge.

**Case Presentation:**

In this case report, an isolated torsion of the right fallopian tube was described in a virgin girl who was treated laparoscopically. The patient presented to the University Hospital of Larisa, in Greece (January 2017) after 5 days of sharp pain in right iliac fossa. Through this period, she looked for medical care in her home country, in Albania. However, further treatment was not available there.

**Conclusion:**

This case manifested that laparoscopy plays an important role in the accurate diagnosis of isolated torsion of the fallopian tube. It thwarts unnecessary delay in treatment and it requires an endoscopy unit. Unfortunately, endoscopy is not commonly the main diagnostic procedure in many countries, including Greece.

## Introduction

Isolated torsion of the fallopian tube is a very rare condition. The reported incidence is 1 in 1,500,000 women (1). It was first described in 1890 (2). It occurs without involvement of the ovaries, as in this case. Predisposing factors for torsion of the fallopian tube are anatomical abnormalities of the tube, abnormal motility of the tube, haematosalpinx, hydrosalpinx, ovarian or para-ovarian masses, previous tubal surgery, trauma, infection, venous congestion in mesosalpinx, sudden changes of body position and pregnancy (3).

## Case Presentation

In University Hospital of Larisa in Greece (January 2017), a 21-year-girl, who had no sexual relationship, was presented to the emergency department with a sharp pain of right iliac fossa for 5 days. Previously, she has been examined in other hospital facilities in Albania, but the patient declined laparotomy. No procedure was performed for her, due to the lack of an endoscopic unit. Eventually she came to University Hospital of Larisa.

There were no bowel or bladder symptoms. Moreover, there was no significant past medical or surgical history.

During the clinical examination, she was afebrile and had tenderness in the right iliac fossa with no guarding or rebound tenderness. On rectal examination, she had tenderness and fullness in the pouch of Douglas (POD) with cervical excitation. A pelvic ultrasound scan demonstrated a normal uterus and ovaries with a hypo-echoic cystic structure measuring 3×3×4 *cm* adjacent to the right ovary within the recto-uterine pouch with avascular focal mural thickening of 1 *cm*. There was no ascites or free fluid in the pouch of Douglas. The appearances suggested torsion of a right ovarian cyst. Laboratory analysis including full blood count, biochemical parameters, CA125 and C reactive protein values and urinary values were normal. A diagnosis of right side torsion of an ovarian cyst was made pre-operatively and emergency laparoscopy was performed.

**Figure 1.**
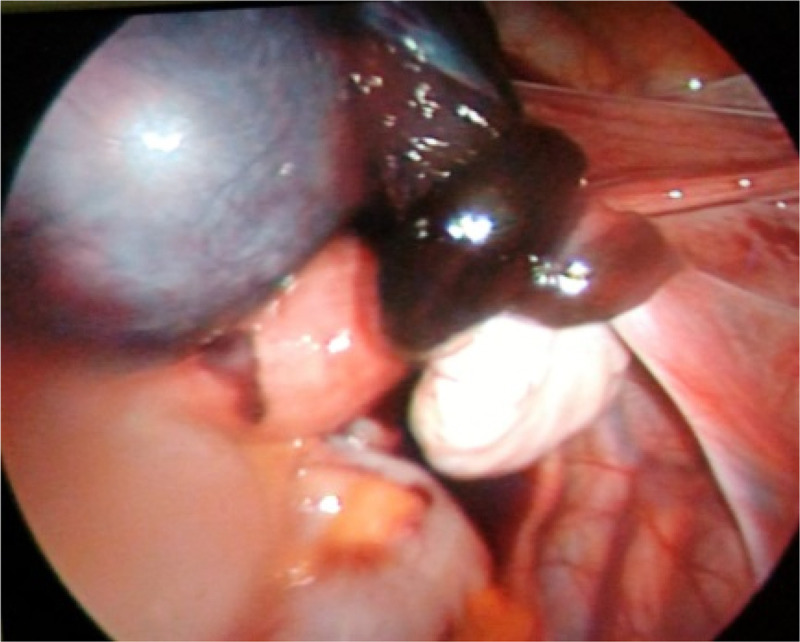
Isolated torsion of the right fallopian tube

The laparoscopy revealed a 3×4 *cm* swollen distal end of right fallopian tube with three twists in the middle (Figure 1). The uterus, both ovaries and left tube, were normal. The rest of the pelvis and liver were normal. A right partial salpingectomy was done laparoscopically.

At first, an attempt was made to preserve the salpinx by restoring the torsion (Twisting) and some time elapsed for blood supply restoration and oedema to recede. Although the torsion was restored, unfortunately the salpinx remained ischemic and the delayed surgical treatment resulted in necrosis.

The post-op course was good since the symptoms disappeared and patient was finally discharged two days after surgery. Microscopic evaluation revealed necrotic and hemorrhagic areas of infiltration with polymorphonuclear leukocytes.

Histopathology report confirmed an infarcted-dilated fallopian tube with ischemia and necrosis.

## Discussion

Isolated torsion of the fallopian tube is a very rare condition among emergency gynecological conditions. However, it is more common as an incident during the reproductive age. A tubal torsion can occur even with an otherwise normal fallopian tube. In this particular case, the patient had a unilateral hydrosalpinx which may have been congenital, as her left tube was normal and there were no other signs of pelvic inflammatory disease (4).

Factors that could influence the occurrence of tubal torsion have been classified into two groups (5). Those two groups are intrinsic and extrinsic. Intrinsic causes include congenital abnormalities, such as excessive length of tube or a spiral course, hydrosalpinx, haematosalpinx, tubal neoplasm and prior surgery particularly tubal sterilization. Extrinsic causes include ovarian and paratubal masses, pregnancy, adhesions, pelvic congestion and sudden body movements. All these factors contribute to the development of tubal torsion, by providing a point of reference around which the tube can twist (6).

Isolated tubal torsion can be managed with either detorsion or simple salpingectomy. Adnexal detorsion has an extremely low risk of thromboembolic events. However, it should be performed as early as possible to avoid irreversible damage to the tissue. The operative approach could be conventional exploratory laparotomy or laparoscopic surgery. In this case, laparoscopy was preferred because not only does it operate as a diagnostic tool but it is also an excellent therapeutic instrument.

In case these techniques are performed in the hospital facilities, the physicians can proceed to investigate underlying causes to prevent recurrent twisting in the same or the other tube.

Although torsion of a fallopian tube more often occurs in the reproductive age group as in this particular case, it has also been reported in medical incidents of pre- menarcheal girls and of perimenopausal women (7, 8).

Most reports describe involvement of the right side (9). This occurs due to the fact that either the mobility of the left tube is limited by the sigmoid colon, or slow venous flow on the right side which may result in congestion, or that more cases of right side pain are surgically explored for suspected acute appendicitis. Isolated recurrent torsion of the fallopian tube has also been reported (10).

The diagnosis of this condition is often delayed because of its scarceness and patients often have prolonged investigations to rule out the more common causes of acute abdomen (11, 12).

However, currently, the number of cases diagnosed preoperatively, through Doppler ultrasonography, computed tomography or magnetic resonance imaging, is increasing (13).

In the particular case, the expeditious diagnosis was based on laparoscopy, even though the ultrasound scan was suggestive of torsion of an ovarian cyst.

This case report highlights the fact that clinically equivocal signs and symptoms should be managed by a minimally invasive procedure such as laparoscopy for immediate and appropriate management (14, 15).

Unfortunately, physicians often hesitate to perform this routine practice due to lack of an endoscopic unit in hospitals.

## Conclusion

Isolated tubal torsion is a rare condition and radiologic investigative tool and endoscopy plays an important role in the accurate diagnosis and avoids unnecessary delay in treatment. All gynecologists should consider this diagnosis in children and women of reproductive age who present with acute lower abdominal pain. Despite this, endoscopy is not currently considered a common diagnostic procedure in many countries including Greece. Although the tube was infarcted in this case, a timely diagnosis and early surgical intervention may preserve the fallopian tube. Even when irreversible damage has occurred, laparoscopic management is recommended.
